# Technological Innovation in Total Knee Arthroplasty: Navigation, Robotics, and Customization

**DOI:** 10.1055/s-0045-1810044

**Published:** 2025-11-04

**Authors:** Marcus Vinicius Malheiros Luzo, Marcio de Castro Ferreira, Alexandre Barbieri Mestriner, Idemar Monteiro de Palma, Carlos Eduardo da Silveira Franciozi, Marcelo Seiji Kubota

**Affiliations:** 1 Knee Surgery Group, Department of Orthopedics and Traumatology, Escola Paulista de Medicina, Universidade Federal de São Paulo, São Paulo, SP, Brazil; 2 Knee Surgery Group, Hospital Maradei, Belém, PA, Brazil

**Keywords:** arthroplasty, replacement, knee, osteoarthritis, knee, prostheses and implants, robot surgery, artroplastia do joelho, cirurgia robótica, osteoartrite do joelho, próteses e implantes

## Abstract

Total knee arthroplasty (TKA) is the gold standard to treat degenerative knee joint conditions that do not improve with conservative treatment, and it yields excellent clinical and functional outcomes. However, a small proportion of patients report some dissatisfaction with their knees after surgery. Recently, TKA underwent significant technological development with the introduction of new biomechanical concepts and the incorporation of techniques to improve surgical procedures. Moreover, the evolution in implant designs optimized for the individual characteristics of the patients enables better surgical customization. The current article presents the concepts, advantages, and limitations to the development of customized implants, as well as TKA surgical techniques involving automation (robotics) and computer-assisted navigation. Although the medical literature showing promising outcomes with new TKA technologies is extensive, studies with longer follow-up and appropriate methodologies are required to contribute to the demonstration of advantages in knee arthroplasty outcomes.

## Resumo

A artroplastia total de joelho (ATJ) é o procedimento padrão-ouro para o tratamento das patologias degenerativas articulares do joelho sem melhora com o tratamento conservador, e resulta em desfechos clínicos e funcionais excelentes. No entanto, uma parcela pequena dos pacientes relata alguma insatisfação com seus joelhos após as cirurgias. Neste contexto, nos últimos anos, o desenvolvimento tecnológico associado a este tratamento tem passado por relevante introdução de novos conceitos biomecânicos e incorporações de tecnologias que buscam o aprimoramento das técnicas cirúrgicas associado à evolução de desenhos de implantes que otimizem as características individuais dos pacientes para permitir melhor customização cirúrgica. O objetivo deste artigo é apresentar conceitos, vantagens e limitações associadas ao desenvolvimento dos implantes customizados, além das técnicas cirúrgicas de ATJ associada à automação (robótica) e à navegação assistida por computação. Ainda que exista ampla literatura médica que demonstra resultados promissores com as novas tecnologias nas cirurgias de ATJ, são necessários estudos com maior seguimento e metodologias adequadas a fim de contribuir com a demonstração de vantagens nos desfechos cirúrgicos nas artroplastias de joelho.

## Introduction

Total knee arthroplasty (TKA) is the gold-standard surgery for the final stage of gonarthrosis. Even though its clinical outcomes are mostly favorable, it is estimated that approximately 10% of the patients remain dissatisfied with the procedure. 1 2

Given the growing demand for TKA and its increasing performance on young adult patients, 3 there is a clear need and search for higher-quality surgical outcomes aiming at implant longevity, a more physiological perception of the joint, better lower limb functionality, and less surgical trauma. 4

In this context, new technological advances, such as specific instrumentation, guides, and computer-assisted (navigated) and/or automated (robotic) surgery, seek better customization. Moreover, these advances consider the physiology and balance of each patient's ligaments to improve three-dimensional surgical planning, increasing the accuracy of alignment and implant positioning. 4

These concepts are being presented and introduced into the arthroplasty care market to improve the mechanical understanding of the lower limb, the articular and functional mapping of the knee, and the precision and surgical reliability to increase patient satisfaction. However, since the incorporation of new technologies usually increases costs, surgeons need to understand TKA innovations to develop a critical sense about their practices and visualize the proper use of these new tools to improve surgical outcomes. 5 6

The present article aims to describe the new technologies in TKA, which involve navigation, robotics, and customized implants, as well as to discuss their current concepts, advantages, and limitations, to help the reader identify how they may contribute to technical development and improve TKA outcomes.

## Customized Implants

The first devices for knee arthroplasty were introduced in the 1970s. Since then, they have undergone several changes, including surgical technique and implant design. 7 An implant is based on the anatomical dimensional parameters of a defined population group, which may lead to inadequacies in the fit of prosthetic components to the joint anatomy in other populations. Studies involving non-Caucasian populations, such as Indians, Chinese, Malaysians, and Koreans, for example, 8 9 10 11 12 have demonstrated diverse anthropometric relationships that differ from those presented by Caucasians. The comparison of these populations showed significant discrepancies in commercial prosthesis models, which can have a direct effect on arthroplasty outcomes. 8 9 10 11 12 Anatomy can vary with gender, ethnicity, biotype, and acquired phenotypic alterations.

Technological development promotes more “personalized” orthopedic therapies focused on the specific conditions of each subject. Although a contemporary trend, therapeutic individualization dates to Hippocrates, who said, “It is more important to know what sort of person has a disease than to know what sort of disease a person has.” 13

Individualized medicine is increasingly present in arthroplasty to obtain better outcomes. Personalized TKA is encouraged by four main pillars: 1) a mechanical axis better adapted to individual biomechanics 14 15 2) anatomical variations that do not fit into predefined implant formats due to joint phenotypic variability; 3) technological evolution enabling better knowledge and intraoperative biomechanical assessment; and 4) commercial pressure from manufacturers to mass-market their new products. 16

Customized components with physiological coating respect geometry and bone limits, avoiding anatomical hyper- or hypodimensioning and reducing the chances of impacts and friction in soft tissues or lack of adequate joint coverage, resulting in more physiological kinematics. However, even when advocating for individualized anatomophysiological joint restoration, it is necessary to reflect that, at times, the natural biomechanics of a given patient may be a causal or risk factor for joint pain and dysfunction.

Customized implants enable personalized definitions of the trochlear groove, favoring patellofemoral kinematics regardless of femoral condylar shapes, which can be manufactured with individual curvature radii. This customization enhances the kinematic benefit of the implant and may contribute to reducing the need for compensation to modify femoral rotation and balance the flexion gap. This condition depends on the absence of ligamentous tension asymmetries requiring soft tissue release to reestablish gap balance. 17 18 19 The physiological and anatomical reestablishment of the posterior condylar curvature increases the flexion and extension gaps. It is worth highlighting the influence of the femoral condylar curvature radius, as its relationship with the posterior capsule also interferes with the dimensioning of the extension gap, a condition the surgeon must keep in mind when dimensioning the offset during arthroplasty planning in patients with flexed or hyperextended knees. 20 21 For a physiological arthroplasty, in which the patient does not perceive their “artificial” knee in daily routines, a specific questionnaire has been developed to measure this outcome. 22

Conceptually, customized TKA should maintain or seek to restore: 1) the perception of a normal joint; 2) “functional” biomechanics (when the native anatomy is not deemed pathological), namely: a) native kinematic axes; b) native orientation of the joint line; c) native tension of soft tissues; and d) native joint kinematics and kinetics during daily activities; 3) adequate mechanical stress transfer between interfaces; 4) native articular range of motion; 5) macro- and microstability of the joint; 6) polyethylene resistance to wear and solid implant fixation, contributing to its survival without activity restriction; and 7) provide fast and complication-free recovery. 2

Customized implants gave rise to new conceptual challenges to the TKA classic technical principles, which are binary between the measured resection and the gap-balancing techniques. 23 As such, Lee et al. 24 proposed a new concept dissociated from the principles of sections perpendicular to the femoral and tibial mechanical axes and called it *kinematic alignment* ; it aims to obtain a joint profile closer to that of the native joint by recreating the three kinematic axes of the knee, which are parallel and perpendicular to the native articular lines. Later, other concepts were introduced, including the inverted kinematic alignment, unrestricted kinematic alignment, functional alignment, and modified kinematic alignment. 24 25 All of these new technical definitions share the manipulation of more “physiological” joint tensions and lines and potential bone section compensations for small asymmetries in ligament tensions. In other words, we now question the concepts previously established by Insall et al. 26 as to whether to restore all native anatomy and ligament laxity. Technological innovations, such as computer-assisted surgery (CAS) and automation, have favored new perceptions and understandings, leading to the current conceptual development for a patient-specific approach, and are promising for optimizing TKA evolution and outcomes. It is worth emphasizing that Krackow, Krena, and Hungerford conceived the concept of anatomical alignment in the 1980s for use with prosthesis preserving the posterior cruciate ligament. This concept was forgotten, largely due to the premature wear of the polished polyethylene used at the time. The advent of new and better materials and the incorporation of navigation-assisted technology and robotics have resulted in a better understanding and use of the anatomical alignment technique. 25

We must mention that the customized TKA concept does not have universal application. In many patients, degenerative arthritic disease can lead to joint and limb deformities and anatomical and biomechanical variations, such as shortening and lengthening of soft tissues and extra- and intraarticular deformities that make it difficult or impossible to identify physiological kinematics to be restored. In addition, analyzing whether the patient's anatomical and physiological mechanical condition can negatively interfere with the implant's longevity requires critical judgment. 27 Therefore, Vendittoli et al. 2 stated that customized TKA does not mean reproducing the patient's anatomy routinely, but offers a more qualified surgical solution to address the disease with a personalized implant technology.

Given all the considerations and advantageous theoretical concepts on customized implants, we still need further clinical evidence to demonstrate superior outcomes of these implants. Müller et al. 28 and Saeed et al. 29 demonstrated that customized TKA did not yield benefits superior to those of conventional implants, and they found a higher early revision rate among personalized implants. Moret et al. 30 did not find significant differences in clinical outcomes between customized and conventional implants. However, personalized implants presented better alignment to the neutral axis of the limb, with better adjustment and positioning.

It is worth emphasizing the need for better research methodologies and study designs in prospective comparisons between personalized and conventional implants to clarify the understanding of the outcomes of customized and classic surgical techniques.

## Computer-Assisted (Navigated) and Automated (Robotic) Arthroplasty

In the late 1970s and early 1980s, surgical and biomechanical concepts on resulting limb alignment and ligament balancing after knee arthroplasty showed greater scientific solidity, favoring the development of better implants and optimizing surgical performance. 26 The early 1990s witnessed the development of the surgical technique for computer-assisted (CA)-TKA. The principle of this technology is the identification of intraoperative anatomical references, such as the centers of the femoral head, of the knee, and of the ankle, providing real-time frontal and lateral views of the mechanical axis of the lower limb for better intraoperative control of the patient's limb axis. The first navigated TKA was performed on January 21, 1997. 31 This technology aims to facilitate the anatomomechanical interpretation of the patient's limb, favoring the technical decisions taken by the surgeon to define the best strategy for joint kinematics and implant positioning.

Navigation-assisted TKAs have evolved technologically and have enabled the surgeon to intraoperatively identify much more than the final resulting limb axis, as they now enable the precise angulation of the femoral and tibial sections, the rotational positioning of the femoral and tibial components, the dimensions of the extension and flexion gaps, and the sizing of the implants. The introduction of CA technology into TKA has enable surgeons to have a broader understanding, better intraoperative joint control, and greater procedural accuracy in planning; this knowledge has contributed to the development of new mechanical concepts of the resulting limb alignment. 25 As such, the concepts of femoral and tibial sections, previously perpendicular to the mechanical axis in conventional mechanical alignment, became more flexible. This flexibility enables joint stability by reducing or avoiding soft tissue releases, that is, enabling a “thin” bone section adjustment compensating for small ligament imbalances through the inclination of the joint line about the mechanical axis, being closer to the anatomy of the native knee. 14

Identifying the potential to increase the accuracy of CA-TKA with surgical precision to perform bone sections, robotic orthopedic surgery has undergone significant development in recent years. In addition, it has managed to increase the quality and practicality of the procedure. 32

Robotic TKA (rTKA) uses software to convert the patient's anatomical records into a three-dimensional knee joint reconstruction, enabling the surgeon to plan the procedure and perform the sections precisely. Therefore, rTKA is a surgical technique uniting CA navigation and robotic automation assistance for the positioning and/or performance of the sections. 14 32 33 34 There are two rTKA subgroups: active (autonomous) rTKA, in which automation is complete and independent of the surgeon's action to perform the planned femoral and tibial sections, and semiactive (semiautonomous) robotic system, which is more widespread globally and currently available in Brazil, in which the surgeon has the overall control of bone resection assisted by the precision of automation through the positioning of bone-cutting guides or by a robotic arm helping the surgeon control the force, range of reach, and direction of the saw blade within programmed limits. 34

In a summarized and succinct manner, the main conceptual difference between CA-TKA and rTKA is that the former is a procedure in which the surgeon positions the guides and performs the sections assisted by computer monitoring. And rTKA is a CA-TKA assisted by automation regarding the positioning of cutting guides and/or the performance of the bone sections. The main benefit of rTKA is the precise and reproducible bone sections due to a robotic interface, regardless of the system. In addition, it enables the measurement of gaps according to the planning of the bone sections and the implant positioning during surgery. 4 The main advantages of rTKA are the accuracy of bone sections as planned, contributing to the ideal implant positioning and joint balance by the exact measurement of the flexion and extension gaps.

There are no absolute indications or contraindications for rTKA or CA-TKA. Nonetheless, these technologies stand out and have an advantage over the conventional technique in patients with diaphyseal bone deformities and intramedullary rods, which hinder the classic surgery.

Patients with joint deformities and bone defects can also benefit from navigation, as this technology enables the surgeon to visualize the multiple possibilities of sections and implant sizing during planning and intraoperatively.

Some automation systems depend on preoperative radiographic examination for the software to interpret the three-dimensional joint reconstruction that will match the anatomical points identified by the surgeon. However, depending on the robotic CA system, the surgery may not require prior radiological mapping, enabling the capture of all anatomical references and spatial identification of the knee and lower limb during the procedure.

It is worth noting that CA-TKA enables precise critical control of many concepts and technical elements relevant to the functional behavior of the knee to promote mechanical and kinematic predictability to the operated limb before and after bone sections, including:

- The posterior sagittal tibial slope to form the flexion gap;
- The degree of rotation of the femoral component to equalize the flexion gap between the medial and lateral femorotibial compartments and potential notching of the anterior femoral cortex;
- The dimensioning and proportionality between the extension and flexion gaps according to the dimensions of the femoral component and the tibial polyethylene;
- The size of the femoral component and its relationship with the dimensioning of the flexion gap;
- The positioning of the femoral implant in the sagittal plane;
- The ability to interchange the femoral and tibial implants;
- The resulting angulation of the lower limb about the mechanical axis per planned adjustments;
- The resulting sagittal of the lower limb in neutral, recurved, or flexed positions;
- The dimensioning of the distal and posterior femoral and proximal tibial sections;
- The height of the joint interline;
- The equalization of the flexion and extension gaps; and
- Polyethylene dimensioning.

The literature 35 36 37 38 presents favorable and promising outcomes regarding the ability of this technology to result in greater precision of the components, soft tissue protection, greater patient satisfaction, short learning curve, ideal ergonomic design, and lower levels of fatigue for the surgeon and surgical team. Other studies have demonstrated clinical outcomes similar to those of the conventional techniques. In a systematic review analyzing more than 6 thousand patients, Onggo et al. 32 concluded that rTKA and conventional TKA are reliable, safe, and yield good outcomes, even though the final limb alignment is more accurate with robotics. A review by Mancino et al. 39 found an advantage for the rTKA group in terms of the initial functional outcomes and radiolucency lines compared to the group submitted to the conventional procedure. They reported no significant differences between the two groups in terms of overall survival, revision rate, and operative time. In their systematic review and meta-analysis of 12 randomized clinical studies involving more than 2 thousand patients, Ruangsomboon et al. 40 concluded that, although rTKA probably results in better radiological accuracy than the conventional technique, this outcome may not have clinical significance.

A recent systematic review by Nogalo et al. 41 explored complications in robotic arthroplasties. The main complications observed were metaphyseal fractures in the fixation holes of the navigation reflector pins, with ∼ 1.4% occurring around the twelfth postoperative week. The incidence rate of infection at the reflection pin and accessory incision sites was of 0.47%. 42 It is worth emphasizing that robotic surgery is safe. Nonetheless, complications may occur and are more related to the surgical technique than a direct consequence of the technology. 43

## Final Considerations

Given the considerations addressed in the current study regarding the purposes of incorporating new technologies and technical concepts into TKA, the advances are promising. Still, further studies with higher-quality methodologies and longer follow-up periods are required to understand the medium and long-term technical advantages regarding the clinical outcomes and, consequently, to enable the development of better techniques and practices for knee arthroplasties.

## References

[1] DeFrance M J Scuderi G R Are 20% of Patients Actually Dissatisfied Following Total Knee Arthroplasty? A Systematic Review of the Literature J Arthroplasty 2023 38 03 594 599 10.1016/j.arth.2022.10.011 36252743

[2] Vendittoli P A Riviere C Hirschmann M T Bini S Why personalized surgery is the future of hip and knee arthroplasty: a statement from the Personalized Arthroplasty Society EFORT Open Rev 2023 8 12 874 882 10.1530/EOR-22-0096 38038379 PMC10714387

[3] Shichman I Roof M Askew N Nherera L Rozell J C Seyler T M Schwarzkopf R Projections and Epidemiology of Primary Hip and Knee Arthroplasty in Medicare Patients to 2040–2060 JB JS Open Access 2023 8 01 e22.00112 10.2106/JBJS.OA.22.00112 PMC9974080 36864906

[4] Batailler C Swan J Sappey Marinier E Servien E Lustig S New Technologies in Knee Arthroplasty: Current Concepts J Clin Med 2020 10 01 47 10.3390/jcm10010047 33375702 PMC7795103

[5] Pagan C A Karasavvidis T Cohen-Rosenblum A R Hannon C P Lombardi A V Jr Vigdorchik J M Technology in Total Knee Arthroplasty in 2023 J Arthroplasty 2024 39(9S2):S54 S59 10.1016/j.arth.2024.07.028 39053667

[6] Walgrave S Oussedik S Comparative assessment of current robotic-assisted systems in primary total knee arthroplasty Bone Jt Open 2023 4 01 13 18 10.1302/2633-1462.41.BJO-2022-0070.R1 36602297 PMC9887337

[7] Liau J J Cheng C K Huang C H Lo W H The effect of malalignment on stresses in polyethylene component of total knee prostheses–a finite element analysis Clin Biomech (Bristol, Avon) 2002 17 02 140 146 10.1016/s0268-0033(01)00109-7 11832264

[8] Vaidya S V Ranawat C S Aroojis A Laud N S Anthropometric measurements to design total knee prostheses for the Indian population J Arthroplasty 2000 15 01 79 85 10.1016/s0883-5403(00)91285-3 10654467

[9] Yue B Varadarajan K M Ai S Tang T Rubash H E Li G Differences of knee anthropometry between Chinese and white men and women J Arthroplasty 2011 26 01 124 130 10.1016/j.arth.2009.11.020 PMC3740371 20149574

[10] Hussain F Kadir M RA Zulkifly A H Anthropometric measurements of the human distal femur: a study of the adult Malay population BioMed Res Int 2013 2013 175056 10.1155/2013/175056 24294597 PMC3835611

[11] Ha C W Na S E The correctness of fit of current total knee prostheses compared with intra-operative anthropometric measurements in Korean knees J Bone Joint Surg Br 2012 94 05 638 641 10.1302/0301-620X.94B5.28824 22529083

[12] Cheng F B Ji X F Lai Y Three dimensional morphometry of the knee to design the total knee arthroplasty for Chinese population Knee 2009 16 05 341 347 10.1016/j.knee.2008.12.019 19230678

[13] Valentino M Pavlica P Medical ethics J Ultrasound 2016 19 01 73 76 10.1007/s40477-015-0189-7 26941884 PMC4762847

[14] MacDessi S J Oussedik S Abdel M P Victor J Pagnano M W Haddad F S The language of knee alignment: updated definitions and considerations for reporting outcomes in total knee arthroplasty Bone Joint J 2023 105-B 02 102 108 10.1302/0301-620X.105B2.BJJ-2022-1345 36722056

[15] Rivière C Lazic S Boughton O Wiart Y Vïllet L Cobb J Current concepts for aligning knee implants: patient-specific or systematic? EFORT Open Rev 2018 3 01 1 6 10.1302/2058-5241.3.170021 29657839 PMC5890125

[16] Saffarini M Hirschmann M T Bonnin M Personalisation and customisation in total knee arthroplasty: the paradox of custom knee implants Knee Surg Sports Traumatol Arthrosc 2023 31 04 1193 1195 10.1007/s00167-023-07385-0 36934205

[17] Müller J H Li K Reina N Telmon N Saffarini M Cavaignac E Sexual and ethnic polymorphism result in considerable mismatch between native trochlear geometry and off-the-shelf TKA prostheses Knee Surg Sports Traumatol Arthrosc 2020 28 12 3871 3878 10.1007/s00167-020-05871-3 32020254

[18] Rivière C Lazic S Villet L Wiart Y Allwood S M Cobb J Kinematic alignment technique for total hip and knee arthroplasty: The personalized implant positioning surgery EFORT Open Rev 2018 3 03 98 105 10.1302/2058-5241.3.170022 29657851 PMC5890135

[19] Zhang Z Liu M Wen X Liu S Zhang G Yang C The relationship between the posterior tibial slope and the sagittal femoral condylar shape: Two circles and ellipses Clin Anat 2020 33 07 1075 1081 10.1002/ca.23543 31880335

[20] Sugama R Kadoya Y Kobayashi A Takaoka K Preparation of the flexion gap affects the extension gap in total knee arthroplasty J Arthroplasty 2005 20 05 602 607 10.1016/j.arth.2003.12.085 16309995

[21] Sriphirom P Raungthong N Chutchawan P Thiranon C Sukandhavesa N Influence of a secondary downsizing of the femoral component on the extension gap: a cadaveric study Orthopedics 2012 35(10, Suppl)56 59 10.3928/01477447-20120919-59 23026254

[22] Ferreira M C Silva G Zidan F F Franciozi C E Luzo M VM Abdalla R J Forgotten Joint Score - Portuguese translation and cultural adaptation of the instrument of evaluation for hip and knee arthroplasties Rev Bras Ortop 2018 53 02 221 225 10.1016/j.rboe.2018.02.006 29911090 PMC6001155

[23] Dennis D A Komistek R D Kim R H Sharma A Gap balancing versus measured resection technique for total knee arthroplasty Clin Orthop Relat Res 2010 468 01 102 107 10.1007/s11999-009-1112-3 19789934 PMC2795818

[24] Lee Y S Howell S M Won Y Y Lee O-S Kinematic alignment is a possible alternative to mechanical alignment in total knee arthroplasty Knee Surg Sports Traumatol Arthrosc 2017 25 11 3467 3479 10.1007/s00167-017-4558-y 28439636

[25] Minoda Y Alignment techniques in total knee arthroplasty J Joint Surg Res 2023 1 01 108 116 10.1016/j.jjoisr.2023.02.003

[26] Insall J N Binazzi R Soudry M Mestriner L A Total knee arthroplasty Clin Orthop Relat Res 1985 192 13 22 3967412

[27] Rivière C Jackson W Villet L Sivaloganathan S Barziv Y Vendittoli P A Specific case consideration for implanting TKA with the Kinematic Alignment technique EFORT Open Rev 2021 6 10 881 891 10.1302/2058-5241.6.210042 34760288 PMC8559564

[28] European Knee Associates (EKA) Müller J H Liebensteiner M Kort N Stirling P Pilot P Demey G No significant difference in early clinical outcomes of custom versus off-the-shelf total knee arthroplasty: a systematic review and meta-analysis Knee Surg Sports Traumatol Arthrosc 2023 31 04 1230 1246 10.1007/s00167-021-06678-6 34432095

[29] Saeed A Z Khaleeq T Ahmed U Correction to: No clinical advantage with customized individually made implants over conventional off-the-shelf implants in total knee arthroplasty: a systematic review and meta-analysis Arch Orthop Trauma Surg 2024 144 03 1331 10.1007/s00402-023-05180-7 38183432

[30] Moret C S Schelker B L Hirschmann M T Clinical and Radiological Outcomes after Knee Arthroplasty with Patient-Specific versus Off-the-Shelf Knee Implants: A Systematic Review J Pers Med 2021 11 07 590 10.3390/jpm11070590 34206259 PMC8304027

[31] Saragaglia D Rubens-Duval B Gaillot J Lateur G Pailhé R Total knee arthroplasties from the origin to navigation: history, rationale, indications Int Orthop 2019 43 03 597 604 10.1007/s00264-018-3913-z 29589088

[32] Onggo J R Onggo J D De Steiger R Hau R Robotic-assisted total knee arthroplasty is comparable to conventional total knee arthroplasty: a meta-analysis and systematic review Arch Orthop Trauma Surg 2020 140 10 1533 1549 10.1007/s00402-020-03512-5 32537660

[33] Liow M H Xia Z Wong M K Tay K J Yeo S J Chin P L Robot-assisted total knee arthroplasty accurately restores the joint line and mechanical axis. A prospective randomised study J Arthroplasty 2014 29 12 2373 2377 10.1016/j.arth.2013.12.010 24439796

[34] Kayani B Konan S Ayuob A Onochie E Al-Jabri T Haddad F S Robotic technology in total knee arthroplasty: a systematic review EFORT Open Rev 2019 4 10 611 617 10.1302/2058-5241.4.190022 31754467 PMC6836078

[35] Khlopas A Sodhi N Sultan A A Chughtai M Molloy R M Mont M A Robotic Arm-Assisted Total Knee Arthroplasty J Arthroplasty 2018 33 07 2002 2006 10.1016/j.arth.2018.01.060 29506926

[36] Fang D M Ritter M A Davis K E Coronal alignment in total knee arthroplasty: just how important is it? J Arthroplasty 2009 24(6, Suppl)39 43 10.1016/j.arth.2009.04.034 19553073

[37] Parratte S Price A J Jeys L M Jackson W F Clarke H D Accuracy of a New Robotically Assisted Technique for Total Knee Arthroplasty: A Cadaveric Study J Arthroplasty 2019 34 11 2799 2803 10.1016/j.arth.2019.06.040 31301912

[38] Song E K Seon J K Yim J H Netravali N A Bargar W L Robotic-assisted TKA reduces postoperative alignment outliers and improves gap balance compared to conventional TKA[Erratum in Clin Orthop Relat Res 2012 Sep;470(9):2627]Clin Orthop Relat Res 2013 471 01 118 126 10.1007/s11999-012-2407-3 22669549 PMC3528918

[39] Mancino F Cacciola G Malahias M-A What are the benefits of robotic-assisted total knee arthroplasty over conventional manual total knee arthroplasty? A systematic review of comparative studies Orthop Rev (Pavia) 2020 12 01 8657 10.4081/or.2020.8657 32913593 PMC7459388

[40] Ruangsomboon P Ruangsomboon O Pornrattanamaneewong C Narkbunnam R Chareancholvanich K Clinical and radiological outcomes of robotic-assisted versus conventional total knee arthroplasty: a systematic review and meta-analysis of randomized controlled trials Acta Orthop 2023 94 60 79 10.2340/17453674.2023.9411 36805771 PMC9941983

[41] Nogalo C Meena A Abermann E Fink C Complications and downsides of the robotic total knee arthroplasty: a systematic review Knee Surg Sports Traumatol Arthrosc 2023 31 03 736 750 10.1007/s00167-022-07031-1 35716186 PMC9958158

[42] Held M B Gazgalis A Neuwirth A L Shah R P Cooper H J Geller J A Imageless robotic-assisted total knee arthroplasty leads to similar 24-month WOMAC scores as compared to conventional total knee arthroplasty: a retrospective cohort study Knee Surg Sports Traumatol Arthrosc 2022 30 08 2631 2638 10.1007/s00167-021-06599-4 33961067

[43] Pagani N R Menendez M E Moverman M A Puzzitiello R N Gordon M R Adverse Events Associated With Robotic-Assisted Joint Arthroplasty: An Analysis of the US Food and Drug Administration MAUDE Database J Arthroplasty 2022 37 08 1526 1533 10.1016/j.arth.2022.03.060 35314290

